# Semiparametric modeling for the cardiometabolic risk index and individual risk factors in the older adult population: A novel proposal

**DOI:** 10.1371/journal.pone.0299032

**Published:** 2024-04-18

**Authors:** Philippe Tagder, Margareth Lorena Alfonso-Mora, Diana Díaz-Vidal, Aura Cristina Quino-Ávila, Juliana Lever Méndez, Carolina Sandoval-Cuellar, Eliana Monsalve-Jaramillo, María Giné-Garriga

**Affiliations:** 1 Fisioterapia, Universidad de Boyacá Sede Tunja, Colombia; 2 Real World Evidence, IQVIA, Belgium; 3 Fisioterapia, Universidad de La Sabana, Campus del Puente del Común, Cundinamarca, Colombia; 4 Fisioterapia, Facultad Ciencias de la Salud- Grupo GIMHUS, Universidad de San Buenaventura-Cartagena, Colombia; 5 Department of Sport Sciences, Faculty of Psychology, Education and Sport Sciences Blanquerna, Universitat Ramon Llull, Barcelona, Spain; 6 Department of Physical Therapy, Faculty of Health Sciences Blanquerna, Universitat Ramon Llull, Barcelona, Spain; Universite de Kinshasa, THE DEMOCRATIC REPUBLIC OF THE CONGO

## Abstract

The accurate monitoring of metabolic syndrome in older adults is relevant in terms of its early detection, and its management. This study aimed at proposing a novel semiparametric modeling for a cardiometabolic risk index (CMRI) and individual risk factors in older adults. Methods: Multivariate semiparametric regression models were used to study the association between the CMRI with the individual risk factors, which was achieved using secondary analysis the data from the SABE study (Survey on Health, Well-Being, and Aging in Colombia, 2015). Results: The risk factors were selected through a stepwise procedure. The covariates included showed evidence of non-linear relationships with the CMRI, revealing non-linear interactions between: BMI and age (p< 0.00); arm and calf circumferences (p<0.00); age and females (*p*<0.00); walking speed and joint pain (p<0.02); and arm circumference and joint pain (p<0.00). Conclusions: Semiparametric modeling explained 24.5% of the observed deviance, which was higher than the 18.2% explained by the linear model.

## Introduction

Cardiovascular diseases (CVD) are the leading cause of morbidity and mortality, accounting for approximately 31% of all deaths worldwide. Several risk factors are involved in the pathogenesis of CVD, including a set of metabolic abnormalities related to ectopic lipid deposition, insulin resistance, and chronic low-grade inflammation [1]. The recognized cardiovascular risk factors (CVRFs) are age, sex, smoking, diabetes, total cholesterol, low-density lipoprotein cholesterol (LDL-C), high-density lipoprotein cholesterol (HDL-C), and blood pressure (BP) [2]. Other CVRFs are family history, obesity, body fat distribution, stress, and socioeconomic status, which also affect the calculated risk [2].

The American College of Cardiology and the American Heart Association (ACC/AHA) established the pooled cohort equation (PCE) model of CVRF to inform the risk of atherosclerotic cardiovascular disease (ASCVD). They advocated its use in clinical decisions for prescribing cholesterol and hypertension-targeted therapies. The PCE includes cardiovascular risk factors, such as age; sex; race; blood pressure; total and HDL cholesterol (dyslipidemias); smoking; diabetes (glycemia levels); and the use of antihypertensive drugs [1, 3]. This group of CVRFs confers an overall risk of CVD that is significantly greater than the sum of the individual risk factors [4]. Therefore, its use for the determination of total risk is relevant in the context of clinical practice.

Metabolic syndrome (MetS) is a consequence of cardiovascular risk, which is it-self a set of CVRF and is associated with an increased risk of multiple chronic diseases, including cancer and acute myocardial infarction [4, 5]. The International Diabetes Federation and the AHA/NHLBI agreed that the presence of three of five risk factors constitutes a diagnosis of MetS; these are the following: elevated waist circumference, elevated triglycerides, reduced HDL-C, elevated blood pressure, and elevated fasting glucose. The prevalence of MetS is rapidly increasing in the Western world. Its health consequences include a two-fold increased risk of developing CVD and type 2 diabetes mellitus, and a 1.5-fold increased risk of all-cause mortality [6].

Although the association between MetS and cardiovascular risk has long been established, there are several shortcomings in the literature when it comes to the older adult population. Challenges in assessing MetS in older subjects include the lack of universally applied diagnostic criteria and divergences among the clinical guidelines, for this age group, for recommended cardiovascular targets. In addition, questions persist as to whether MetS is a valid construct for older adults, as certain authors have suggested that the overall prognostic value of MetS in this group may not be greater than the individual risk factors being taken separately [7]. Likewise, increasing age, particularly >65, constitutes a risk factor for the prevalence of metabolic syndrome [8].

According to Global Burden of Disease (GBD) estimates for 2019, “ischemic heart disease and stroke were the top-ranked causes of Disability-Adjusted Life-Years (DALYs) in both the 50–74-year and 75-years-and-older age groups”. Both diseases are related to cardiometabolic risk in the older adult population, and the risk could be reduced through engaging with a healthier lifestyle choice. The GBD also indicates the rise in the risk factors of CVD, such as high blood sugar, high body mass index [BMI], elevated cholesterol, among others [9]. The GBD contends that modifiable risk factors, such as blood pressure, high LDL-C, high BMI, tobacco consumption, dietary habits, and low physical activity, have also risen in the last decade bringing consequences into the major prevalence of CVD and to complications that are derived of itself [3].

Effectively monitoring the level of risk throughout a lifes may allow the implementation of strategies to screen, prevent, and control CVD and MetS risk. Furthermore, providing estimations of the risk profile of the older adult population could be useful in identifying the subjects/subgroups with a higher prevalence of cardiometabolic risk.

Cardiometabolic risk index (i.e., the CMRI score) was previously proposed by Eisenmann 2008 [10] and used by DeBoer et al. [11, 12] and Ramírez-Vélez et al. [7]; this index is based on the International Diabetes Federation statement [6]. The CMRI has been suggested as an accurate method by which to detect overall metabolic changes [12]. The CMRI score is defined as CMRI.score =“z-Waist”+”z-triglycerides”-“z-HDL”+”z-glucose”+”z-SBP”+”z-DBP” where the function z-score is the standardization of each variable, ‘Waist’ is the waist circumference, SBP/DBP are systolic/diastolic blood pressure, and HDL/LDL are the high/low density lipoproteins.

An increase in any of the z-components of the CMRI could result in no impact on the clinical binary diagnosis but would impact the continuous CMRI. A higher CMRI is indicative of a less favorable metabolic syndrome profile, such that the index could be used to diagnose, screen, or monitor the evolution of the cardiometabolic risk [6, 7, 10–12]. High risk was de-fined as CMRI ≥ mean (CMRI)+SD (CMRI); this means that the higher the value in the CMRI, then the higher the cardiovascular risk [7].

By using multivariate semiparametric and parametric regression models, this study aimed to propose a novel semiparametric modeling for the cardiometabolic risk index and their associated covariates in the older adult population. This was achieved using a secondary analysis cross-sectional sample of Colombian older adults between April and September 2015. Our hypothesis was that the continuous predictors’ indexes would have a non-linear relation with the CMRI. This assumption was tested when the semi-parametric model was compared to the parametric model.

## Materials and methods

A secondary analysis was conducted on the cross-sectional nationally representative SABE study, the Survey on Health, Well-Being, and Aging in Colombia. The data recollection process was developed in 2015 with the protocol of Albala et al. [8]. The primary data of this study were conducted in accordance with the guidelines in the Declaration of Helsinki. This study was also approved by the Ethics Committee of the Universidad de Caldas (protocol ID CBCS-021-14) and the Universidad del Valle (protocol IDs 09–014 and O11-015). All individuals included in the study provided written and informed consent for participation. Colombian Older adults (i.e., 60 years or older, both male and female subjects) were evaluated with physical and psychological self-reported questionnaires and tests. The SABE study was initially devised by the Pan American Health Organization/World Health Organization (PAHO/WHO) as a multicenter survey by which to profile the living and health conditions of older adults in seven urban centers in Latin America and the Caribbean. The biomarker sampling component involved collecting samples from a population of 4,545 older adults residing in 86 municipalities across the country. Among these samples, 79.3% (3,607) were obtained from municipalities with convenient accessibility, while 20.1% (938) were from municipalities with limited access.

This sample should be considered as a random subsample drawn from the overall SABE Survey sample, with replacements of the final sampling units, as previously specified. The primary reasons for not including certain individuals in the sample were related to medication use, recent food consumption, alcohol consumption on the day preceding the sample collection, and non-compliance with fasting requirements.

Sample estimation for biomarker measurement considered the population of Colombian women aged 60 and older residing in Colombian territory. The parameters used for determining the sample size included the following: Proportion (P): 0.07; Design effect (Deff): 1.2; Relative Standard Error (Esrel): 0.065; Percentage of non-response: 20%. This resulted in a calculated sample size of n = 3,771, which was then adjusted to account for the non-response rate.

Based on this sample size and the chosen cities for data collection, it was estimated that for biomarker measurement, two out of every five surveyed individuals needed to be selected. The key principles underlying this sampling scheme included random participant selection for biomarker measurement, systematic selection based on the sampling fraction relative to the general sample, maximized geographical distribution within the constraints of available resources, and representation in major cities at the regional and national levels. The data depuration process included a cleaned and organized approach as follows: We used the observations without missing values for the selected predictors (complete cases). We had 2104 observations in the biomarker sub-sample (triglycerides, HDL, glucose) of the SABE survey. The outlier observations that were considered typographical errors were (1) people with a height less than 50 cm or greater than 266 cm; (2) a calf/arm/waist circumference that was equal to 0; (3) people with a gait speed that was equal to 0; and (4) people weighing less than 27 kg or greater than 635 kg. After filtering such missing data and outlier observations, the final sample size was 1524. Other important predictors, such as dynamometry (right and left) and the chair stand test, were not included in the model because they had more than 35% missing of their data in the sub-sample.

The older adults’ characteristics were used to predict the association with the CMRI; of the response variables, five predictors were considered continuous, and 35 categorical. The predictors that were included were as follows:

- Continuous predictors: age; arm and calf circumference; average walking speed; and body mass index (BMI).
- Categorical predictors: antihypertensive, diabetes, cholesterol and triglycerides medicine; balance test; money; food; shopping; drugs; transport and phone use (the instrumental activities of daily living); risk fall; and demographics, such as gender, transport, smoke, alcohol, socioeconomic status, ethnicity, living alone, difficulty in walking 400 meters, needing help to walk 400 meters, shortness of breath, dizziness, back pain, weakness, tiredness, nausea, respiration thirst, joint pain, difficulty in sleeping, cancer, chronic obstructive pulmonary disease, arthritis, osteoporosis, psychiatry, emotional areas, brain stroke, and physical activity.

### Data analysis

A multivariate Lasso modeling procedure was performed; this was achieved by avoiding univariable screening since the latter can miss important variables that are only important after adjusting for other variables [13]. The semiparametric model was informed by the previous stepwise parametric model, which provided a selection of only the categorical predictors. A mixed-model-based penalized splines model regression was conducted using mgcg:gam [14], following previously described models [14, 15]. Three steps were used (1) the optimal non-parametric structure for the thin plate regression splines (bs = “tp”) was selected using restricted maximum likelihood (REML) penalization; (2) the inclusion of interactions between predictors was tested; and (3) an additional extra penalty was conducted with the final model to each term, such that it could be penalized to zero using the Lasso method. The presented confidence interval was 95% point-wise estimations. In each model, the model assumptions were checked, as were the need to use an additional Box–Cox transformation for the models.

Three types of interactions were conducted, i.e., by a factor-by-curve (when a continuous variable interacts with a categorical variable) fashion, in a isotropicthin plate (when two continuous variables interact with the same scale), and in a tensor-product-smooth approach (when the two continuous variables interact without sharing the same scale). In the case of nested models, the selection of the best interaction was based on the Aikake information criteria (AIC) but was contrasted with the likelihood ratio test (LRT) under the F distribution assumption. For the final model comparison, only the AIC and the Bayesian information criterion (BIC) were used to select between the non-nested models.

The final analysis of this study include the CMRI cut point, which have been suggested by the literature [7] (mean (CMRI)+sd(CMRI)) to categorize the subject as high or low cardio-metabolic risk, whereby the original scales’ cut point is 3.12. If we consider such CMRI cut points to predict the MeTS diagnosis in the considered sample, the discriminative properties are quite poor (sensitivity = 0.28, specificity = 0.97, accuracy = 0.68). This is especially the case when recognizing healthy subjects (sensitivity<0.5, i.e., worse than guessing). However, can the discriminative properties of the CMRI be improved? The answer is absolutely: the use of an optimal cut point model is the best approach, and it is one that we will present in the results section.

All statistical analyses were performed with R software (version 4.2.2) (R Core Team (2022)) using the mgcg package [14]. A mixed model-based penalized splines model regression was conducted using mgcg::gam [14], following previously described models [15].

## Results

### Parametric modeling phase

Simple linear parametric and non-parametric non-penalized linear regressions were explored with each continuous predictor, where the predictors arm/calf circumference and BMI showed the clearest non-linear pattern (not shown). This naïve exploration suggests that a non-parametric penalized approach can be useful. With respect to this, three linear multiple parametric models are presented: model “A”, model “B”, and model “C”.

Model A is a parametric linear regression (A) with all predictors. This model explains a statistically significant and moderate proportion of variance (R^2^ = 0.24, F(78, 1438) = 5.72, *p* < .001, adj. R^2^ = 0.20) using 1517 observations and 86 parameters, and which delivers an AIC = 7641. The normality assumptions for A were not satisfactory in the diagnostic plots and in the formal test (Kolmogorov–Smirnov test *p*< 0.0013 for the residuals); this suggests the need for a transformation to improve the normality assumptions of the model residuals. In the observed population, the CMRI score has an average of -0.14, with a minimum value of -10.05, a maximum of 18.15, and a standard deviation = 3.26. The original Box–Cox transformation requires a positive response value; as such, for simplicity, we use CMRI_score+15 (minimum = 4.95, maximum = 33.15, sd = 3.26, mean = 14.86).

Model B is a Box–Cox transformation (B) with all predictors; in such a model the Box–Cox method for the response variable CMRI_score+15 (with a full model) provides an estimation of:

λ=0.41(1571observations,AIC=7641,81parameters).

With such transformation there are no more concerns regarding the regression assumptions (i.e., the model explains a statistically significant and moderate proportion of variance (R^2^ = 0.20, F(74, 1442) = 4.91, p < .001, adj. R^2^ = 0.16)). This is such because we obtained similar diagnostics plot and a Kolmogorov–Smirnov test p-value for a model that uses the optimal λ (Kolmogorov–Smirnov test p> 0.1264) and an approximation value of λ = 1/3 (Kolmogorov–Smirnov test *p* = 0.1264). As such, we decided to use the latter transformation to facilitate the interpretability:

$$\mathrm{Boxcox}\;\mathrm{transformation}=\frac{{\left(CMRI_{score}+15\right)}^{\lambda }-1}{\lambda }\approx \frac{\sqrt[{3}]{CMRI_{score}+15}-1}{1/3}=3\sqrt[{3}]{CMRI_{score}+15}-2$$

All the predictions made with the transformed response variable were back transformed, such that the estimates can be interpreted in the original units. The back-transformation formula (for all the models) is:

$$exp\left(\frac{log(1+\lambda 3CMRI.bc_{score})}{\lambda }\right)-15\approx exp(3log(1+CMRI.bc_{score}/3))-15$$

Model C used a stepwise selection, using previous Box–Cox transformation (BoxCoxTr:brt); the selected predictors were: sex, BMI, physical activity (yes not), calf and arm circumference, joint pain (yes not), socioeconomic and psychiatric (using a cross-validation at a 5-fold level). Despite some of the continuous variables not being selected in this stepwise procedure, we kept it for the non-parametric phase. Our hypothesis is that such continuous variables could be meaningful under a non-parametric assumption. Moreover, the final variables that will be included in the non-parametric models are arm and calf circumference, sex, BMI, physical activity, joint pain, socioeconomic status, psychiatric diagnoses, age, walk speed and, antihypertensive, diabetes, cholesterol and triglyceride medicines. The linear model fit of such a model provides an AIC = 2190 (1517 observations, 16 parameters). This model explains a statistically significant and moderate proportion of variance (R^2^ = 0.17, F (15, 1501) = 20.65, *p* < .001, adj. R^2^ = 0.16). An additional Box–Cox exploration with the latter model (stepwise selection) does not change the Box–Cox estimates for λ. In addition, the model assumptions were double-checked, presenting no concerns (Kolmogorov–Smirnov test *p* = 0.1108).

The multi-collinearity was explored in the continuous variables, using the correlation, and no multicollinearity was observed.

### Non-parametric modeling phase

Using the procedures comparison of different models we present three possible models (S1 Appendix) the second one is the one that is preferred for prediction (based on AIC criteria 2114) and for estimation (based on BIC criteria 2346). The lambda Box–Cox interaction for the selected model provided a Box–Cox lambda that was close to the identity (1.11), which suggests that no further update was required. This final model was fitted with an additional Lasso penalization, which improves the fit, and no terms were thus dropped. Where f_k,1≤k≤6 are the smooth functions: a) for one predictor and b) for two predictors. Where i = 1..1524 are the number of observations. Now, by substituting the estimates predictors for the categorical variables (Table 1).

**Table 1:** Parametric and non–parametric estimates for the selected model with the best BIC.

***Parametric coefficient***	estimate	std.error	statistic	p.value	
(Intercept)	4.33	0.03	134.23	0.00	
Gender Male	0.25	0.03	8.53	0.00	
Physical Activity Yes	-0.10	0.03	-3.76	0.00	
Joint pain Yes	-0.09	0.03	-3.47	0.00	
Cancer Yes	-0.07	0.05	-1.39	0.16	
Psychiatric Yes	0.12	0.06	2.16	0.03	
Etnia Unknown	0.05	0.03	1.57	0.12	
Etnia White	0.02	0.03	0.79	0.43	
Area Rural	0.04	0.03	1.26	0.21	
Alcohol Yes	-0.01	0.04	-0.28	0.78	
Smoke Yes	-0.01	0.03	-0.56	0.58	
***Approximate significance of smooth terms***			statistic	p.value	edf
s(Age): Gender Female			1.89	0.00	2.42
s(Age):Gender Male			0.00	0.71	0.00
s(Circ_arm):Joint painNo			0.71	0.00	1.38
s(Circ_arm):Joint painYes			0.76	0.00	2.05
s(Circ arm,Circ calf)			0.64	0.00	8.69
s(Walk_speed):Joint_painNo			1.28	0.01	3.51
s(Walk_speed):Joint_painYes			0.07	0.22	0.44
s(BMI)			0.62	0.00	1.87
te(Age,BMI)			1.17	0.00	3.60

**Fig 1:**
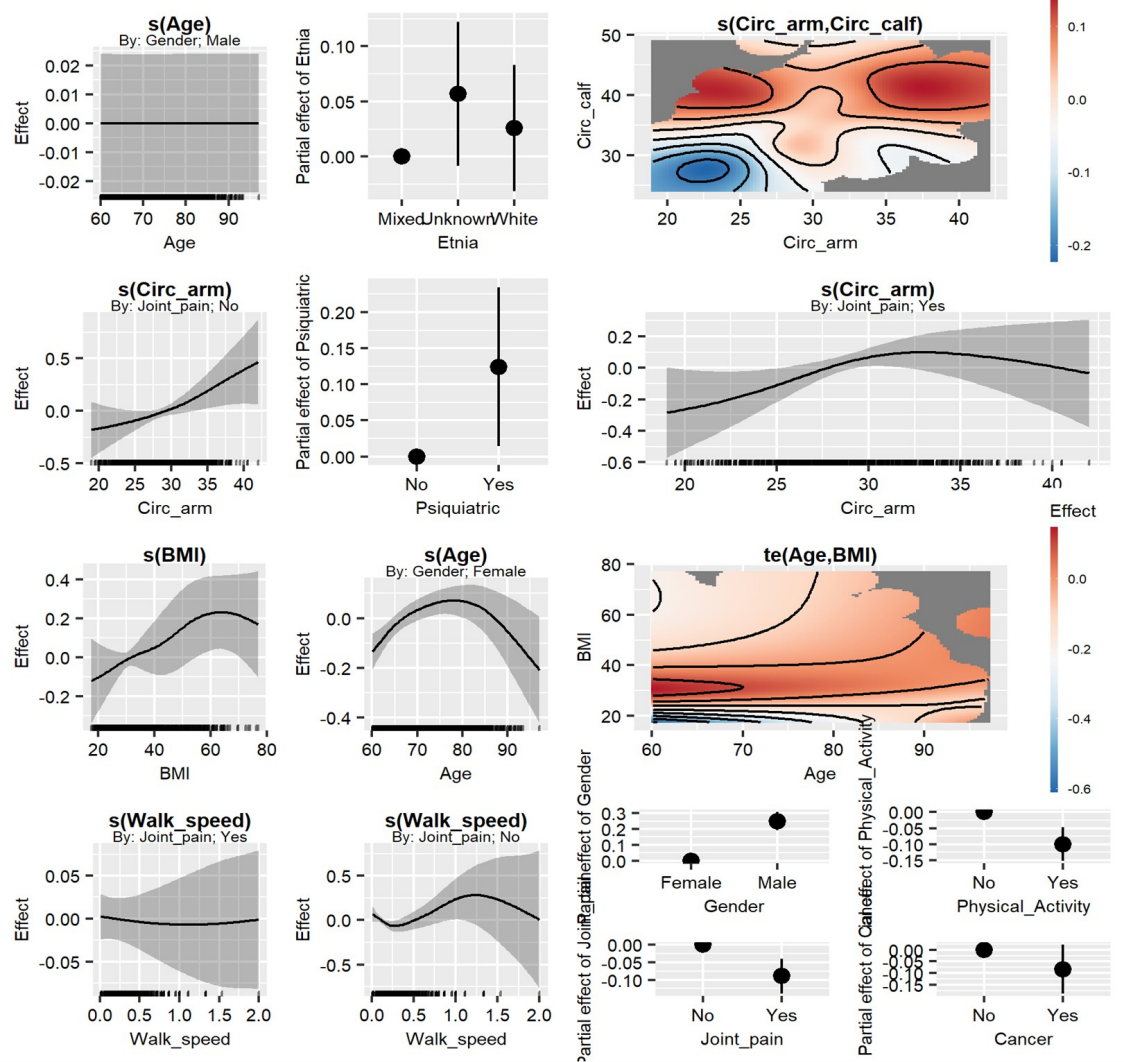
Semiparametric selected model (GAM.Inter.2). The component smooth functions for the continuous predictors.

**Fig 2:**
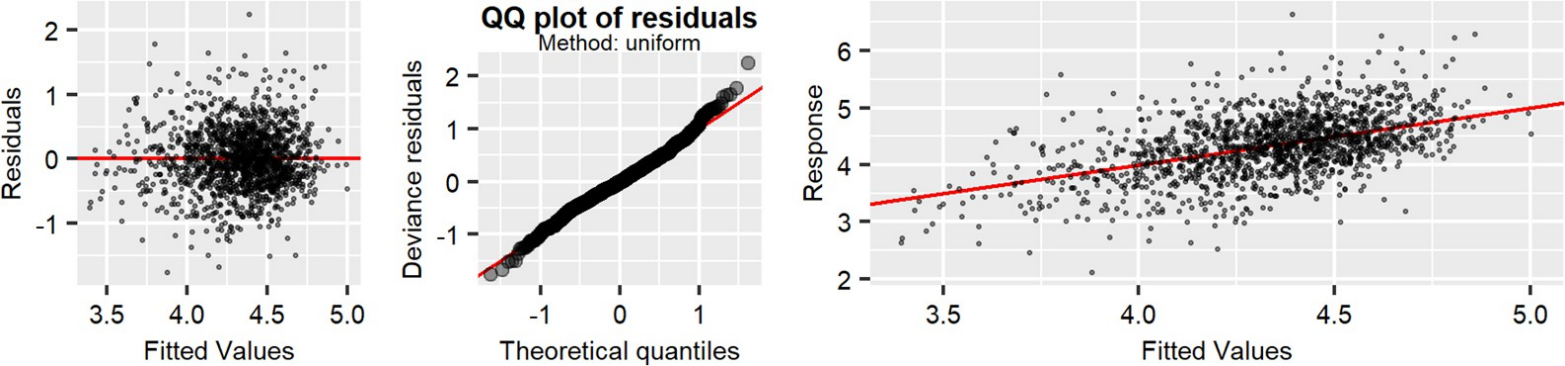
The assumption’s diagnosis: qq plot from residuals plots, residuals/predicted plots, and worm plots. The predictors expression are: (Age by = Gender, k = 10) + s(arm calf circumff, k = 15) + s(walk speed by = gender, k = 10) + s(BMI2, k = 15) + Gender + Excercise * 3 week. The shaded region indicates approximately 0.95 pointwise confidence intervals.

The estimated coefficients from model chosen for the categorical variables are substituted, but the rest of the coefficient were too complex to be expressed as an equation; as such, the recommendation was always to see the non-linear effect estimation (Figs 1 and 2), but they can be described in a qualitative fashion, as follows: The continuous interaction between the circumferences was modeled as an isotropic-thin plate (the recommended option Wood 2017 for measures that share the same unit of measure), but the BMI and Age was modeled as a tensor product smooth (usual recommendation [14] for predictors that do not share the same unit of measure).

In the selected model, we observed evidence of the non-linear relationships for all the continuous variables (Table 1 and Fig 1). For female subjects, an inverted U-shape was observed for Age. For the male subjects, a linear trend for age and walking speed was presented. In female subjects, the CRMI decrease with Walking speed until 0.5; it then increased to 1 (a J-shape response). It is remarkable that, for males, no dependency exists between Age and the CMRI; however, in general, the risk in males is higher than females (Table 1). The CMRI increase with Age in females until 80, which then most likely decrease. The CMRI increases linearly with the BMI for both sexes. Between 60–70 years, the CMRI increase between 25 <BMI <50, which then decrease. After 80 years, the CMRI increase with any BMI that is bigger than 25. The CMRI increase with the arm circ in the absence of joint pain. However, with the presence of joint pain, an increase occurs until 30 cm. The male subjects’ habits of no exercise or walking, show that the absence of Joint pain and Psychiatric conditions are thus riskier (S2 Appendix).

The number of spline basis dimension functions in the selected model was checked, showing that each smooth parametrization terms is sufficient (S3 Appendix). All fitted models used REML with a penalty-based model selection, which assures us that the degree of freedom for each parameter is optimal. The optimization of the parametrization uses mixed model-based penalized splines with REML smoothing parameter selection.

Additionally, the model assumptions were checked using diagnostic plots, the quantile-quantile plot shows that the assumed normal error distribution is reasonable, and the plot of the residuals against the fitted values shows no indication of heteroscedasticity.

The clinical use of this semiparametric model allows for comprehension of the behavior to the interactions of variables continuous and categorical in relation to CMRI (Fig 1). The linear models usually determine the function of one-by-one variables instead of the non-linear model presenting interactions between them. An example of that is shown in Figs 1 and 2 where the CMRI in the female sex combined with age shows a specific distribution, the high risk is evident in the range of 75–80 years and decreases after that age.

Finally, authors to recommend include assessment of variables like walk speed, arm circumference, or calf circumference in relation with normative values of older adults with cardiometabolic risk.

### The CMRI optimal threshold for diagnosis

**Fig 3:**
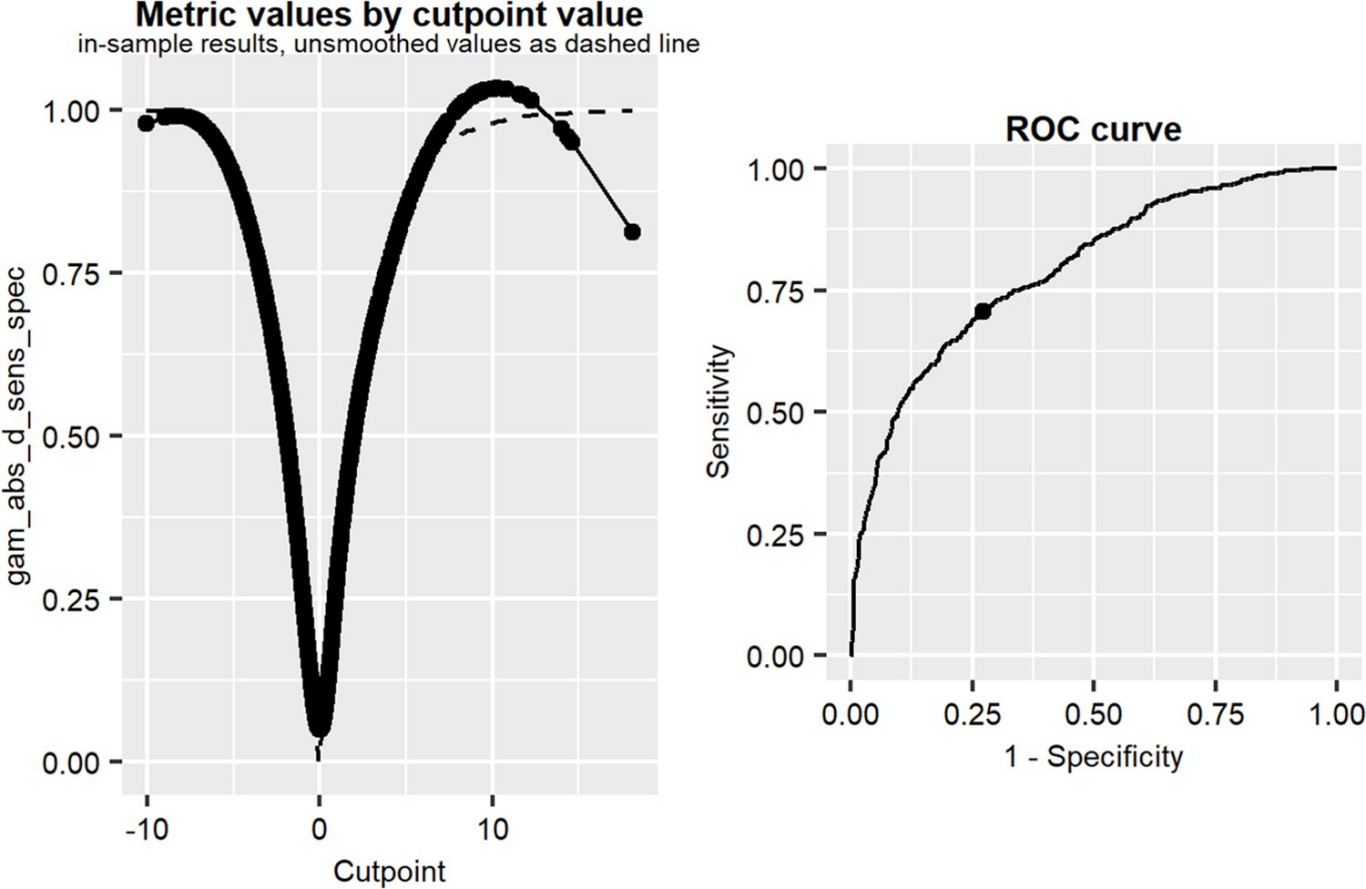
Left: the metric values by cut point value. Right: the ROC curve. Left: The estimation of the optimal CMRI cut point, using a GAM method. Right: the receive operator curve for CMRI, predicting the MeTS diagnosis.

The CMRI proposed by Ramirez-Velez 2019, presented in Methods Section, used a cut point of 3,2. In using the suggested cut point risk in the population sample, the Box-Cox scale is 4.876 = mean CMRI+ SD(CMRI) = 4.334+0.542. In Fig 3 (right), we can observe the histogram of cases and controls using the classical definition and two cut points.

**Fig 4:**
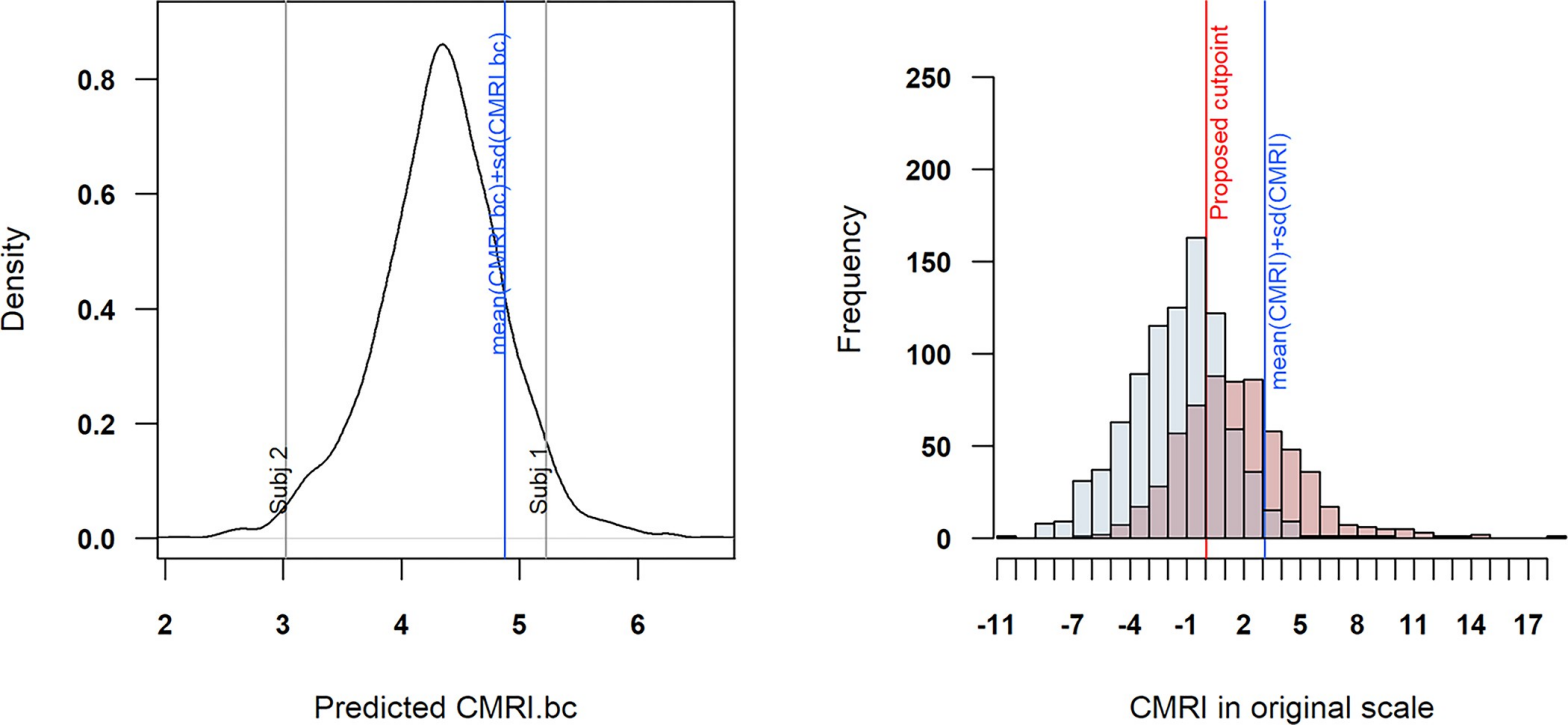
Left: the density function of the predicted CMRI scores. Right: the CMRI in original scale. Left: the density function of the predicted CMRI scores, showing the previous pro–posed CMRI cut point (blue line). Right: a histogram for the observed CMRI in original scale, colored by cases and controls according to the MeTS diagnosis; the new estimated cut point for the CMRI is shown (redline). Please note that both figures are not presented in the same scale.

The connection between the CMRI and the classical MeTS diagnosis is explored using a generalized additive model (GAM). An attractive feature of using a GAM method is that it allows the calculation of the optimal cut point without the need to choose a fixed method by which to select the optimal cut point (as is the case in Youden, AUC, etc.). The discriminative estimation for such GAM cut point models are sensitivity = 0.71, specificity = 0.73, accuracy = 0.72, with an optimal cut point of 0.02 (Fig 4), which is better than the previous cut point.

## Discussion

This study proposes a semiparametric model to estimate the interactions of different covariates with the CRMI, revealing non-linear interactions between different covariates as Age female gender, calf and arm circumference. Also, this semiparametric modeling explained 24.57% of the observed deviance, which is higher than the 17.27% explained by the linear model. The semiparametric models provided a more flexible framework that could improve the assumptions’ violation and goodness of fit but remained clinically easy to interpret.

Despite the calculation of the cut point that is proposed by the literature being straightforward (CMRI = 3.12), it was not shown to be a sensitive enough option [7]. With this study, the authors recommend using a robust estimation (being based on GAM models, for example) of the cut point (CMRI = 0.02) in order to transform the CMRI into a binary classification of the subjects. The model cut point model require a binary diagnosis response with the CMRI as the predictor. The binary MeTS diagnosis was calculated using the criteria set by the International Diabetes Federation.

Modeling the continuous predictors in a non-parametric framework allowed us to show a significant association of variables that, in a parametric framework, did not show a good predictive effect, such as age, that is coincident with previous studies [16–18]. Additionally, this study found a negative association between the CMRI and physical activity, such as those reported by Paley and Johnson [19]. A positive association was found between 1) the CMRI and BMI, as reported by Al-Bachir [20], 2) the arm Shi et al. [21], and 3) the calf circumferences [22]. Regarding sex and joint pain, a negative association with the CMRI was found.

If there is any discrepancy between our non-parametric model for the CMRI and the classic MeTS model, it will be explainable because the CMRI does not include variables such as medicines consumption. However, the CMRI includes different variables that are related to the cardiovascular elements; in addition, it is more relevant and sensible than the classic MeTS risk classification. Regarding joint pain and the sex variables, the authors found a positive association between the male sex and joint pain. Moreover, for the male sex, a high CMRI was found, while the female sex had a high risk, according to MeTS. This could be supported by the fact that the two models of risk are different, as we de-scribed above (see Supporting information).

Even though the association between the CMRI with walking speed is not intuitive, the similar non-linear J-shaped relationship has been reported previously [23]. Regardless of the similarities, the direct comparison is not possible because our response is integrated (the CMRI). Furthermore, in previous studies this had been reported separately (as waist, glucose, SBP, DBP, and HDL). Other studies have also shown associations between walking speed and [24, 25], but in a linear way. As mentioned before, the semiparametric models are still not clinically easy to interpret, and this kind of modeling process is just obtaining force in the statistical analysis of risk.

We found a certain degree of agreement in the direction of the associations with previous studies for the individual risk factor of sex. Previous studies have shown that, in Colombia, MetS is more common in older women than men [26, 27]. Additionally, we found evidence of a negative association between the CMRI and joint pain, which is not supported by the existing [28, 29]. Possible reasons that could explain our results could be the absence of potential confounders in our model or in the additional treatments that such populations received—which we did not consider.

A limitation of the current study is that the back-transformation was only used to compute the predictive estimates, but not to update the model estimates. As the used transformation was conducted in a cubic square root approach, no effect on the direction of the effect (for both parametric and non-parametric terms) was expected. However, an effect on the magnitude should be expected. Additionally, this study has not included the predictive values of the semiparametric model.

To be able to use a regression model in the clinical context, validation with external data is usually required, which confirms the predictive capacity of the variables. This ensures the application by clinicians and police-makers, however, this was not within the scope of this article, so it is recommended for future research. Our article tries to present the benefits of nonlinear modeling, and to raise awareness in future research on how the predictive capacity of the variable can be improved if a nonlinear parameterization of the predictors is allowed.

The semiparametric modeling better explained the observed deviance than the linear model, which is a common finding in the use of semiparametric modeling; this is because semiparametric models [30, 31] are robust to-ward assumption deviation tan parametric models, and they can potentially provide more power in the parameters’ estimation when non-linearity between response and predictors is observed. The clinical use of this predictive model is the applicability of the screening scores with continuous variables that could explain the risk of MeTS.

## Conclusions

The semiparametric modeling explained 24.57% of the observed deviance, which is higher than the 17.27% explained by the linear model. To further improve our methods, a similar model with a bigger sample size could be applied to allow the inclusion of additional predictors. The principal strength of this semiparametric modeling was that it presented a cut point by which to determine the risk of MeTS in the a posteriori of data analysis, thus allowing a better prognosis of the CMRI.

## Supporting information

S1 Appendix Model performances: The AIC, the BIC, the percentage of explained deviance, the R2, the AIC, and the BIC. (PDF)

S2 Appendix Parametric model versus the semiparametric model. (PDF)

S3 Appendix Predictors of the model. (PDF)
