# Correction: Semiparametric modeling for the cardiometabolic risk index and individual risk factors in the older adult population: A novel proposal

**DOI:** 10.1371/journal.pone.0321893

**Published:** 2025-04-02

**Authors:** Philippe Tadger, Margareth Lorena Alfonso-Mora, Diana Díaz-Vidal, Aura Cristina Quino-Ávila, Juliana Lever Méndez, Carolina Sandoval-Cuellar, Eliana Monsalve-Jaramillo, María Giné-Garriga

The first author’s name is spelled incorrectly. The correct name is: Philippe Tadger. The correct citation is: Tadger P, Alfonso-Mora ML, Díaz-Vidal D, Quino-Ávila AC, Méndez JL, Sandoval-Cuellar C, et al. (2024) Semiparametric modeling for the cardiometabolic risk index and individual risk factors in the older adult population: A novel proposal. PLoS ONE 19(4): e0299032. https://doi.org/10.1371/journal.pone.0299032.
