# Supplementary material for: Semiparametric modeling for the cardiometabolic risk index and individual risk factors in the older adult population: A novel proposal
Source: PLoS One. 2024 Apr 18;19(4):e0299032. doi: 10.1371/journal.pone.0299032 (PMC11025852; doi:10.1371/journal.pone.0299032)
Supplement: S1 Appendix — (PDF) [file pone.0299032.s001.pdf]

A1. Model performances: the AIC, the BIC, the percentage of explained deviance, the R2, the AIC, and the BIC.

Model	Predictors	AIC	BIC	Deviance explained	R.square
**GAM0**	Age + Circ_arm + Circ_calf + Walk_speed + BMI + Gender + Physical_Activity + Joint_pain + Cancer + Psiquiatric + Etnia + Area + Alcohol + Smoke	2207	2298	0.1708	0.1625
**GAM1a**	s(Age) + Circ_arm + Circ_calf + Walk_speed + BMI + Gender + Physical_Activity + Joint_pain + Cancer + Psiquiatric + Etnia + Area + Alcohol + Smoke	2207	2298	0.1711	0.163
**GAM1a1**	s (Age, by = Physical_Activity) + Circ_arm + Circ_calf + Walk_speed + BMI + Gender + Physical_Activity + Joint_pain + Cancer + Psiquiatric + Etnia + Area + Alcohol + Smoke	2206	2296	0.171	0.163
**GAM1a2**	s (Age, by = Gender) + Circ_arm + Circ_calf + Walk_speed + BMI + Gender + Physical_Activity + Joint_pain + Cancer + Psiquiatric + Etnia + Area + Alcohol + Smoke	2198	2298	0.1779	0.169
**GAM1b**	Age + s(Circ_arm) + Circ_calf + Walk_speed + BMI + Gender + Physical_Activity + Joint_pain + Cancer + Psiquiatric + Etnia + Area + Alcohol + Smoke	2189	2294	0.1834	0.174
**GAM1b1**	Age + s (Circ_arm, by = Joint_pain) + Circ_calf + Walk_speed + BMI + Gender + Physical_Activity + Joint_pain + Cancer + Psiquiatric + Etnia + Area + Alcohol + Smoke	2179	2289	0.1898	0.18
**GAM1b2**	Age + s (Circ_arm, by = Gender) + Circ_calf + Walk_speed + BMI + Gender + Physical_Activity + Joint_pain + Cancer + Psiquiatric + Etnia + Area + Alcohol + Smoke	2195	2317	0.1833	0.1728
**GAM1c**	Age + Circ_arm + s(Circ_calf) + Walk_speed + BMI + Gender + Physical_Activity + Joint_pain + Cancer + Psiquiatric + Etnia + Area + Alcohol + Smoke	2198	2299	0.1776	0.1686
**GAM1c1**	Age + Circ_arm + s (Circ_calf, by = Physical_Activity) + Walk_speed + BMI + Gender + Physical_Activity + Joint_pain + Cancer + Psiquiatric + Etnia + Area + Alcohol + Smoke	2201	2316	0.1787	0.1687
**GAM1c2**	Age + Circ_arm + s (Circ_calf, by = Gender) + Walk_speed + BMI + Gender + Physical_Activity + Joint_pain + Cancer + Psiquiatric + Etnia + Area + Alcohol + Smoke	2198	2312	0.1803	0.1704
**GAM1d**	Age + Circ_arm + Circ_calf + s(Walk_speed) + BMI + Gender + Physical_Activity + Joint_pain + Cancer + Psiquiatric + Etnia + Area + Alcohol + Smoke	2205	2301	0.1732	0.1647
**GAM1d1**	Age + Circ_arm + Circ_calf + s (Walk_speed, by = Joint_pain) + BMI + Gender + Physical_Activity + Joint_pain + Cancer + Psiquiatric + Etnia + Area + Alcohol + Smoke	2201	2307	0.1771	0.1678
**GAM1d2**	Age + Circ_arm + Circ_calf + s (Walk_speed, by = Gender) + BMI + Gender + Physical_Activity + Joint_pain + Cancer + Psiquiatric + Etnia + Area + Alcohol + Smoke	2205	2304	0.1735	0.1648
**GAM1e**	Age + Circ_arm + Circ_calf + Walk_speed + s (BMI, k = 15) + Gender + Physical_Activity + Joint_pain + Cancer + Psiquiatric + Etnia + Area + Alcohol + Smoke	2139	2259	0.2126	0.2021
**GAM1e1**	Age + Circ_arm + Circ_calf + Walk_speed + s (BMI, k = 15, by = Joint_pain) + Gender + Physical_Activity + Joint_pain + Cancer + Psiquiatric + Etnia + Area + Alcohol + Smoke	2148	2293	0.213	0.2005

Model	Predictors	AIC	BIC	Deviance explained	R.square
**GAM1e2**	Age + Circ_arm + Circ_calf + Walk_speed + s (BMI, k = 15, by = Gender) + Gender + Physical_Activity + Joint_pain + Cancer + Psiquiatric + Etnia + Area + Alcohol + Smoke	2144	2292	0.2158	0.2031
**GAM1f**	Age + s (Circ_arm, Circ_calf) + Walk_speed + BMI + Gender + Physical_Activity + Joint_pain + Cancer + Psiquiatric + Etnia + Area + Alcohol + Smoke	2184	2337	0.1957	0.1825
**GAM1f1**	Age + te(Circ_arm, Circ_calf, k = 15) + Walk_speed + BMI + Gender + Physical_Activity + Joint_pain + Cancer + Psiquiatric + Etnia + Area + Alcohol + Smoke	2192	2349	0.1924	0.1799
**GAM1f2**	Age + s(Circ_arm) + s(Circ_calf) + ti(Circ_arm, Circ_calf, k = 15) + Walk_speed + BMI + Gender + Physical_Activity + Joint_pain + Cancer + Psiquiatric + Etnia + Area + Alcohol + Smoke	2188	2355	0.1963	0.1832
**GAM1g**	Circ_arm + Circ_calf + Walk_speed + Age * BMI + Gender + Physical_Activity + Joint_pain + Cancer + Psiquiatric + Etnia + Area + Alcohol + Smoke	2208	2304	0.1715	0.1715
**GAM1g1**	te(Age, BMI) + Circ_arm + Circ_calf + Walk_speed + BMI + Gender + Physical_Activity + Joint_pain + Cancer + Psiquiatric + Etnia + Area + Alcohol + Smoke	2141	2255	0.2105	0.2105
**GAM1g2**	Circ_arm + Circ_calf + Walk_speed + ti(Age) + ti(BMI) + ti(Age, BMI) + Gender + Physical_Activity + Joint_pain + Cancer + Psiquiatric + Etnia + Area + Alcohol + Smoke	2142	2250	0.209	0.209
**GAM.Inter.1**	s (Age, by = Gender) + s (Circ_arm, Circ_calf) + s (Walk_speed, by = Joint_pain) + s (BMI, k = 15) + Gender + Physical_Activity + Joint_pain + Cancer + Psiquiatric + Etnia + Area + Alcohol + Smoke	2119	2337	0.2414	0.2249
**GAM.Inter.2**	s (Age, by = Gender) + s (Circ_arm, by = Joint_pain) + s (Circ_arm, Circ_calf) + s (Walk_speed, by = Joint_pain) + s (BMI, k = 15) + Gender + Physical_Activity + Joint_pain + te(Age, BMI) + Cancer + Psiquiatric + Etnia + Area + Alcohol + Smoke	2114	2346	0.2468	0.2296
**GAM.Inter.3**	s (Age, by = Physical_Activity) + s (Circ_arm, by = Joint_pain) + s (Circ_arm, Circ_calf) + s(Walk_speed) + s (BMI, k = 15) + Gender + Physical_Activity + Joint_pain + te(Age, BMI) + Cancer + Psiquiatric + Etnia + Area + Alcohol + Smoke	2128	2342	0.236	0.22

Parametric Model with the CMRI

The parametric model (plm) or the linear model were presented as a point of reference in order to be compared with the advantages of the semiparametric model. The models with all the same variables as GAM.Inter.2 and without interactions were plm2 <- lm(CMRI.bc ~ Age*Gender+Circ_arm*Joint_pain +Circ_arm*Circ_calf + Walk_speed*Joint_pain+ BMI +Gender+ Physical_Activity + Joint_pain + Age*BMI + Cancer + Psiquiatric + Etnia + Area + Alcohol + Smoke ,data = DF) plm3 <- lm(CMRI.bc ~ Age+Circ_arm+ Circ_calf+BMI +Gender+ Physical_Activity + Walk_speed+Joint_pain + Cancer + Psiquiatric + Etnia + Area + Alcohol + Smoke ,data = DF).

Comparison of the models.

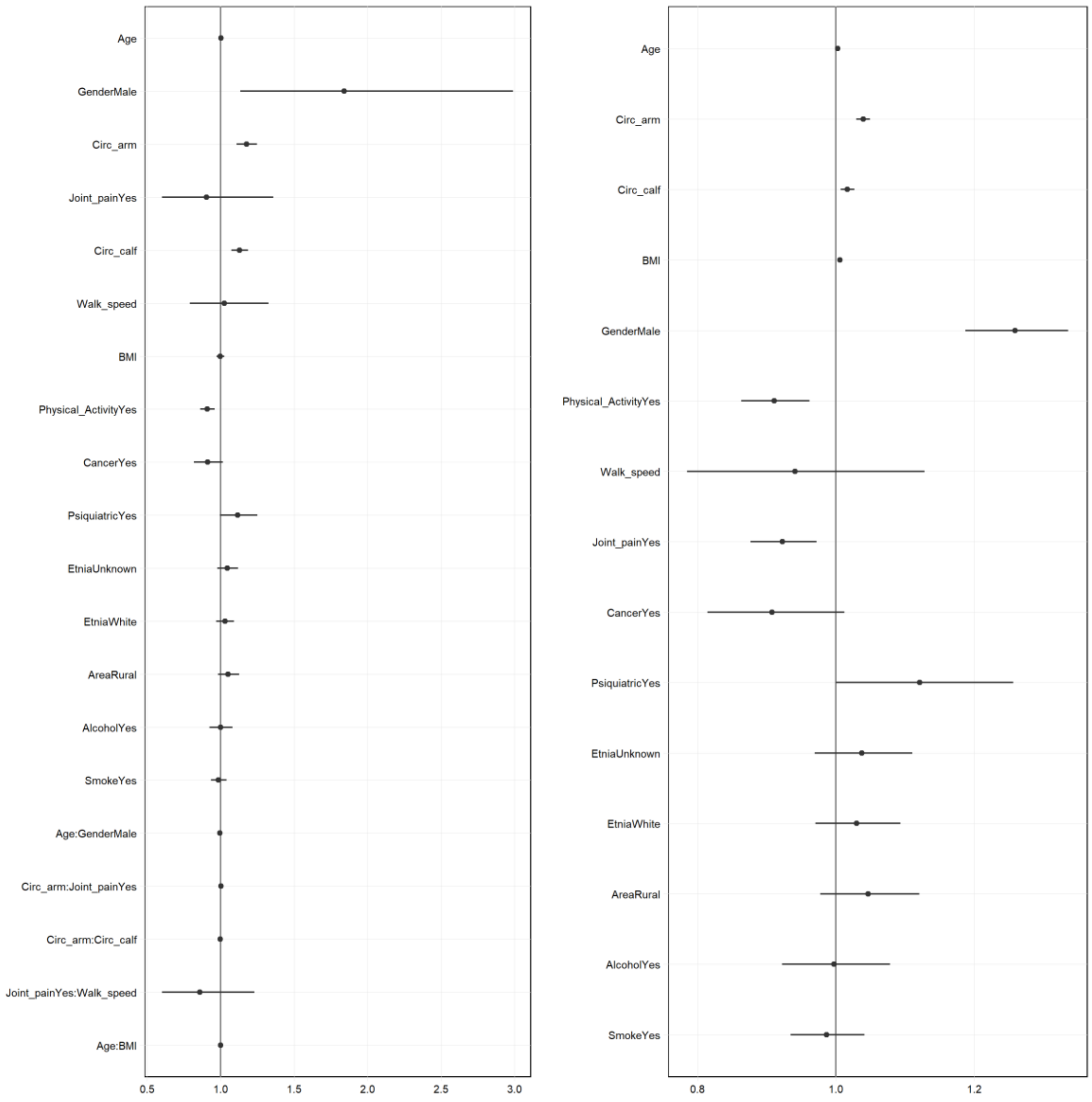

A2. Parametric model versus the semiparametric model.

	Model 1	Model 2
**(Intercept)**	-1.1850	2.2829 ***
	(1.0207)	(0.2081)
**Age**	0.0009	0.0025
	(0.0066)	(0.0019)
**GenderMale**	0.6099 *	0.2304 ***
	(0.2476)	(0.0301)
**Circ_arm**	0.1611 ***	0.0386 ***
	(0.0298)	(0.0049)
**Joint_painYes**	-0.1015	-0.0801 **
	(0.2080)	(0.0264)
**Circ_calf**	0.1210 ***	0.0167 ***
	(0.0257)	(0.0049)
**Walk_speed**	0.0231	-0.0607
	(0.1321)	(0.0924)
**BMI**	-0.0027	0.0059 ***
	(0.0139)	(0.0015)
**Physical_ActivityYes**	-0.0959 ***	-0.0931 ***
	(0.0274)	(0.0275)
**CancerYes**	-0.0932	-0.0967
	(0.0552)	(0.0554)
**PsiquiatricYes**	0.1088	0.1142 *
	(0.0579)	(0.0581)
**EtniaUnknown**	0.0437	0.0370
	(0.0344)	(0.0346)
**EtniaWhite**	0.0281	0.0296
	(0.0301)	(0.0302)
**AreaRural**	0.0496	0.0454
	(0.0347)	(0.0349)
**AlcoholYes**	-0.0006	-0.0029
	(0.0399)	(0.0398)
**SmokeYes**	-0.0150	-0.0135
	(0.0276)	(0.0276)
**Age:GenderMale**	-0.0055	
	(0.0035)	
**Circ_arm:Joint_painYes**	0.0015	
	(0.0071)	
**Circ_arm:Circ_calf**	-0.0036 ***	
	(0.0009)	
**Joint_painYes:Walk_speed**	-0.1524	
	(0.1827)	
**Age:BMI**	0.0001	
	(0.0002)	
**N**	1517	1517
**R2**	0.1828	0.1711
***** p < 0.001; ** p < 0.01; * p < 0.05.**		

A3. Predictors of the model

	k'	edf	k-index	p-value
s(Age):GenderFemale	9	2.42	1.03	0.85
s(Age):GenderMale	9	0.0005	1.03	0.9
s(Circ_arm):Joint_painNo	9	1.38	1.01	0.54
s(Circ_arm):Joint_painYes	9	2.05	1.01	0.62
s(Circ_arm,Circ_calf)	29	8.69	0.98	0.12
s(Walk_speed):Joint_painNo	9	3.51	1.01	0.65
s(Walk_speed):Joint_painYes	9	0.435	1.01	0.68
s(BMI)	14	1.87	0.99	0.36
te(Age,BMI)	23	3.60	1.02	0.73
