# Supplementary material for: Semiparametric modeling for the cardiometabolic risk index and individual risk factors in the older adult population: A novel proposal
Source: PLoS One. 2024 Apr 18;19(4):e0299032. doi: 10.1371/journal.pone.0299032 (PMC11025852; doi:10.1371/journal.pone.0299032)
Supplement: S2 Appendix — (PDF) [file pone.0299032.s002.pdf]

A2. Parametric model versus the semiparametric model.

	Model 1	Model 2
(Intercept)	-1.1850	2.2829 ***
	(1.0207)	(0.2081)
Age	0.0009	0.0025
	(0.0066)	(0.0019)
GenderMale	0.6099 *	0.2304 ***
	(0.2476)	(0.0301)
Circ_arm	0.1611 ***	0.0386 ***
	(0.0298)	(0.0049)
Joint_painYes	-0.1015	-0.0801 **
	(0.2080)	(0.0264)
Circ_calf	0.1210 ***	0.0167 ***
	(0.0257)	(0.0049)
Walk_speed	0.0231	-0.0607
	(0.1321)	(0.0924)
BMI	-0.0027	0.0059 ***
	(0.0139)	(0.0015)
Physical_ActivityYes	-0.0959 ***	-0.0931 ***
	(0.0274)	(0.0275)
CancerYes	-0.0932	-0.0967
	(0.0552)	(0.0554)
PsiquiatricYes	0.1088	0.1142 *
	(0.0579)	(0.0581)
EtniaUnknown	0.0437	0.0370
	(0.0344)	(0.0346)
EtniaWhite	0.0281	0.0296
	(0.0301)	(0.0302)
AreaRural	0.0496	0.0454
	(0.0347)	(0.0349)
AlcoholYes	-0.0006	-0.0029
	(0.0399)	(0.0398)
SmokeYes	-0.0150	-0.0135
	(0.0276)	(0.0276)
Age:GenderMale	-0.0055	
	(0.0035)	
Circ_arm:Joint_painYes	0.0015	
	(0.0071)	
Circ_arm:Circ_calf	-0.0036 ***	
	(0.0009)	
Joint_painYes:Walk_speed	-0.1524	
	(0.1827)	
Age:BMI	0.0001	
	(0.0002)	
N	1517	1517
R2	0.1828	0.1711

*** $p < 0.001$; ** $p < 0.01$; * $p < 0.05$.
