# Supplementary material for: Semiparametric modeling for the cardiometabolic risk index and individual risk factors in the older adult population: A novel proposal
Source: PLoS One. 2024 Apr 18;19(4):e0299032. doi: 10.1371/journal.pone.0299032 (PMC11025852; doi:10.1371/journal.pone.0299032)
Supplement: S3 Appendix — (PDF) [file pone.0299032.s003.pdf]

A3. Predictors of the model

	k'	edf	k-index	p-value
s(Age):GenderFemale	9	2.42	1.03	0.85
s(Age):GenderMale	9	0.0005	1.03	0.9
s(Circ_arm):Joint_painNo	9	1.38	1.01	0.54
s(Circ_arm):Joint_painYes	9	2.05	1.01	0.62
s(Circ_arm,Circ_calf)	29	8.69	0.98	0.12
s(Walk_speed):Joint_painNo	9	3.51	1.01	0.65
s(Walk_speed):Joint_painYes	9	0.435	1.01	0.68
s(BMI)	14	1.87	0.99	0.36
te(Age,BMI)	23	3.60	1.02	0.73
